# Supplementary material for: HRT Atlas v1.0 database: redefining human and mouse housekeeping genes and candidate reference transcripts by mining massive RNA-seq datasets
Source: Nucleic Acids Res. 2020 Jul 14;49(D1):D947–55. doi: 10.1093/nar/gkaa609 (PMC7778946; doi:10.1093/nar/gkaa609)
Supplement: gkaa609_Supplemental_Files [file gkaa609_supplemental_files.zip › Supplementary_Figure 1_.docx]

**HRT Atlas v1.1 database: redefining human and mouse housekeeping genes and candidate reference transcripts by mining massive RNA-seq datasets**

BidossessiWilfried Hounkpe^1^, Francine Chenou^1^, Franciele Lima^1^, Erich Vinicius de Paula^1,2^

Affiliations: ^1^ School of Medical Sciences, University of Campinas, Campinas, SP, Brazil; ^2^ Hematology and Hemotherapy Center, University of Campinas, Campinas, SP, Brazil


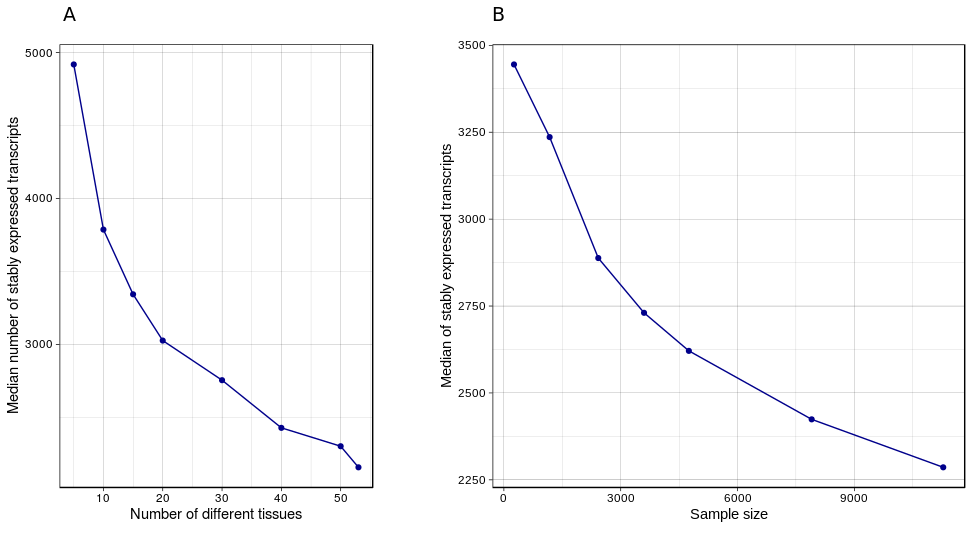


Figure S1: Simulation of the impact of tissue diversity and samples size.

Simulations using GTEx dataset based on random sampling and 100 permutations have been performed to detect the median number of stably expressed transcripts. Our results showed that tissue type diversity (A) and samples size (B) can affect the prediction of HK genes/transcripts. In both simulations the number of transcripts that fulfilled HRT Atlas criteria decreases as the number of tissue types (A) or sample size increases (B).
