# Supplementary material for: HRT Atlas v1.0 database: redefining human and mouse housekeeping genes and candidate reference transcripts by mining massive RNA-seq datasets
Source: Nucleic Acids Res. 2020 Jul 14;49(D1):D947–55. doi: 10.1093/nar/gkaa609 (PMC7778946; doi:10.1093/nar/gkaa609)
Supplement: gkaa609_Supplemental_Files [file gkaa609_supplemental_files.zip › Supplementary_Table2.pdf]

HRT Atlas v1.0 database: redefining human and mouse housekeeping genes and candidate reference transcripts by mining massive RNA-seq datasets

Bidossessi Wilfried Hounkpe1, Francine Chenou1, Franciele Lima1, Erich Vinicius de Paula1,2
Affiliations: 1 School of Medical Sciences, University of Campinas, Campinas, SP, Brazil; 2 Hematology and Hemotherapy Center, University of Campinas, Campinas, SP, Brazil

geNorm stability metrics across 12 different tissues based in RNA-seq datasets

Legend

Newly described reference transcripts

Commonly used reference genes

Gene level expression of the selected reference transcripts (included all of their coding and non-protein coding transcripts)

Rank	Artery Aorta	Brain Cerebellar Hemisphere	Brain Cerebellum
1	ENST00000292807	ENST00000358704	ENST00000316509
1	ENST00000322535	ZBTB18 *	ENST00000447750
3	ENST00000353555	ENST00000378609	ENST00000378609
4	ENST00000418115	ENST00000447750	ENST00000309311
5	ENST00000175091	ENST00000316509	ENST00000375882
6	ENST00000373795	ENST00000309311	ENST00000334478
7	ENST00000309311	ENST00000356674	ENST00000257013
8	ENST00000263645	ENST00000373232	ENST00000360472
9	ENST00000368719	ENST00000250894	ENST00000356674
10	ENST00000216181	ENST00000371646	ENST00000371646
11	LAPTM4A	PPA1	HPRT1
12	HPRT1	PGK1	RTL8C
13	TBP	HPRT1	TBP
14	SRSF4	TBP	PGK1
15	BSG	VAMP2	CSNK2B
16	RHOA	HSP90AB1	VAMP2
17	GUSB	HNRNPA2B1	PEA15
18	S100A6	PPIA	GNB1
19	PPIA	GUSB	HNRNPA2B1
20	PGK1	ACTB	GUSB
21	SF3B2	RPS18	HSP90AB1
22	RPS18	EEF2	PPIA
23	EEF2	GAPDH	RPS18
24	MYH9	TFRC	GDI1
25	AP2M1	GNB1	ACTB
26	CD81	GDI1	TFRC
27	YWHAZ	RPLP0	GAPDH
28	GAPDH	B2M	PFDN5
29	ACTB	MAPK8IP3	EEF2
30	TFRC	YWHAZ	YWHAZ
31	B2M		RPLP0
32	RPLP0		B2M

Rank	Brain Cortex	Breast Mammary Tissue	Cervix Ectocervix
1	ENST00000257013	ENST00000265062	PCBP1*
1	ENST00000357156	ENST00000382581	ENST00000418115
3	ENST00000447750	ENST00000175091	ENST00000357156
4	ENST00000382581	ENST00000330720	NDUFA1*
5	ENST00000037243	ENST00000577035	ENST00000409614
6	ENST00000356674	ENST00000371646	ENST00000309311
7	ENST00000577035	ENST00000356674	ENST00000376630
8	ENST00000360472	ENST00000394936	ENST00000412585
9	ENST00000309311	ENST00000395850	ENST00000376809
10	ENST00000371646	ENST00000309311	DYNLRB1
11	DYNLRB1	LAPTM4A	ENST00000368719
12	MRFAP1	HPRT1	HPRT1
13	HPRT1	MRFAP1	TBP
14	TBP	HSP90AB1	RHOA
15	GABARAPL2	TBP	HLA-E
16	RTL8C	KDELR1	GUSB
17	PGK1	HNRNPA2B1	HLA-A
18	PEA15	PGK1	TFRC
19	HSP90AB1	RAB7A	ACTB
20	GABARAP	GABARAP	HLA-B
21	HNRNPA2B1	GUSB	EEF2
22	GUSB	EEF2	SERF2
23	RPS18	PPIA	B2M
24	PPIA	PSAP	S100A6
25	GDI1	RPS18	PGK1
26	GAPDH	CD59	PPIA
27	EEF2	TFRC	RPS18
28	TFRC	YWHAZ	RPLP0
29	ACTB	GAPDH	GAPDH
30	RPLP0	B2M	YWHAZ
31	YWHAZ	ACTB	
32	B2M	RPLP0	

Rank	HEK293	PANC1	RKO
1	GHITM*	GHITM*	ENST00000233468
1	ENST00000262225	ENST00000262193	ENST00000301764
3	ENST00000373365	ENST00000321301	ENST00000290541
4	ENST00000321301	ENST00000278412	ENST00000438607
5	ENST00000328848	ENST00000368003	ENST00000223641
6	ENST00000373232	ENST00000322535	ENST00000558815
7	ENST00000299300	ENST00000221265	ENST00000272227
8	NOP10	ENST00000345519	ENST00000309311
9	ENST00000262193	ENST00000262225	SF3B6
10	ENST00000299767	ENST00000295702	ENST00000316448
11	PSMB1	PSMB1	ENST00000371646
12	ENST00000253039	UFC1	PDIA6
13	GLO1	SSRP1	SEC61B
14	HPRT1	TBP	HSP90AB1
15	EIF2S3	HPRT1	TBP
16	TMED2	TOMM5	RPL28
17	TOMM5	CLTA	TMA7
18	TBP	SF3B2	GUSB
19	PPA1	GUSB	PPIA
20	PGK1	SSR2	TFRC
21	ACTB	PGK1	DDB1
22	GUSB	PAF1	CALR
23	CCT2	PPIA	GAPDH
24	PPIA	TFRC	EEF2
25	B2M	TMED2	HPRT1
26	GAPDH	ACTB	PGK1
27	TFRC	GAPDH	RPLP0
28	HSP90B1	YWHAZ	ACTB
29	RPS18	RPLP0	B2M
30	YWHAZ	B2M	PSMB4
31	RPLP0	RPS18	YWHAZ
			RPS18

Rank	SKBR3	Stomach	Whole Blood
1	ENST00000237530	ENST00000265062	PCBP1*
1	ENST00000371646	ENST00000332556	ENST00000378609
3	ENST00000433473	ENST00000356674	ENST00000417647
4	ENST00000264645	ENST00000546939	ENST00000418115
5	ENST00000309311	ENST00000300026	ENST00000222305
6	ENST00000262213	LAMP1	ENST00000375882
7	ENST00000394936	ENST00000394936	ENST00000237654
8	ENST00000315758	ENST00000309311	ENST00000295702
9	ENST00000236671	ENST00000371646	ENST00000356674
10	TBP	ENST00000353555	ENST00000309311
11	ENST00000368719	PPIB	HPRT1
12	PSAP	HPRT1	TBP
13	GUSB	TBP	CCNI
14	HPRT1	HSP90AB1	IK
15	HSP90AB1	PGK1	RHOA
16	PGK1	RAB7A	CSNK2B
17	TFRC	HNRNPA2B1	HNRNPA2B1
18	CASC3	BSG	PGK1
19	RPLP0	EEF2	GUSB
20	PPT1	PPIA	GNB1
21	TRAM1	RPS18	SSR2
22	RPN2	GUSB	USF2
23	EEF2	PSAP	EEF2
24	PPIA	TFRC	PPIA
25	B2M	YWHAZ	RPS18
26	GAPDH	CD63	YWHAZ
27	S100A6	GAPDH	TFRC
28	CTSD	RPLP0	ACTB
29	ACTB	B2M	B2M
30	MDH2	ACTB	GAPDH
31	YWHAZ		RPLP0
32	RPS18		
